# Early screening of gestational diabetes mellitus using hemoglobin A1C: Revising current screening guidelines

**DOI:** 10.22088/cjim.10.1.16

**Published:** 2019

**Authors:** Babak Pezeshki, Hossein Chiti, Peyam Arasteh, Saeedeh Mazloomzadeh

**Affiliations:** 1 Zanjan Metabolic Diseases Research Center, Zanjan University of Medical Sciences, Zanjan, Iran; 2 Department of Foreign Languages and Linguistics, Shiraz University, Shiraz, Iran; 3 Social Determinants of Health Research Center, Zanjan University of Medical Sciences, Zanjan, Iran

**Keywords:** Diabetes, Gestational, Early screening, Hemoglobin A1C, Fasting blood glucose

## Abstract

**Background:**

HbA1C has been a known predictor and diagnostic test for diabetes type 2. However, this test has not yet been widely studied in GDM and more importantly no cutoff point has been defined for HbA1C in GDM. We investigated the efficacy of screening during first and second trimester of pregnancy and defined appropriate cutoff points according to HbA1C and FBS for predicting maternal GDM among women with none to minimal previously known risk factors.

**Methods:**

This is a prospective multi-centered cohort study. Individuals were evaluated at first trimester, at 20-24 weeks and 24-28 weeks of gestation. GDM tests were done during visits and accuracy of each of these measurements was evaluated.

**Results:**

Overall, 356 entered the study, among which 25 individuals and 30 individuals developed GDM during 20-24 and 24-28 weeks of gestation, respectively. HbA1C measured during first trimester at a cut-off of 5.35% predicted GDM of 24-28 weeks with an accuracy of 85.6%, sensitivity of 80%, and specificity of 80%. Furthermore, at a cut-off of 5.75% measured at 20-24 weeks, HbA1C predicted GDM with an accuracy of 94.5%, sensitivity of 97%, and specificity of 96%.

**Conclusion:**

Screening programs during first trimester and at 20-24 weeks of pregnancy, using HbA1C, can significantly aid in the early prediction of GDM, even among women with no to minimal previously known risk factors, which shows a need for revision in current guidelines.

Gestational diabetes mellitus (GDM) is the most common metabolic disease of pregnancy which has shown an increasing trend over the past years (1). GDM has always been defined as any degree of glucose intolerance during any period of pregnancy, however the 2016 American Diabetes Association (ADA) guidelines defined GDM as a disease of the second and third trimester of pregnancy and any glucose intolerance before this time to be considered overt diabetes (2, 3). The condition is associated with multiple complications for both the mother and the neonate (born from the mother with GDM), some of which include fetal death, fetal macrosomia, dystocia, neonatal hypoglycemia, acute distress syndrome, eclampsia and preeclampsia, increased cesarean section and etc. (4, 5). Considering the existing guidelines on screening for GDM, some studies have shown that despite appropriate control of GDM during third trimester of pregnancy, mothers with GDM still show complications associated with the disease such as fetal macrosomia.

This shows that the patient may have had uncontrolled blood glucose prior to the diagnosis of the condition during the third trimester of pregnancy which has affected the fetus and resulted in complications (6, 7). Accordingly, early diagnosis of the condition poses a great dilemma. Moreover, those who do not present with any known risk factors for GDM, remain to be highly undetected during pregnancy. 

Recently tests to predict GDM have been a subject of much attention. Among the predictors of diabetes is HbA1C, which has been a known predictor and diagnostic test for diabetes type 2 (8). However, this test has not yet been widely studied in GDM and more importantly no cutoff point has been defined for HbA1C in GDM, especially among those who present with no to minimum previously known risk factors for diabetes. Considering the overwhelming effects of GDM on maternal and neonatal health and the urgent need for early diagnosis and control of maternal glucose levels, we hypothesized that HbA1C may be important for the prediction of GDM. In this study, we evaluated the efficacy of HbA1C measured during the first and second trimester of pregnancy for the prediction of GDM, furthermore, we defined appropriate cutoff points according to HbA1C to predict occurrence of GDM.

## Methods

**Study settings and patients: **This is a prospective multi-centered cohort study conducted in seven health care centers affiliated to Zanjan University of Medical Sciences, Zanjan, Iran. A total of 432 pregnant women between the ages of 18 to 35 years old were considered for inclusion in the study. All women who had the following criteria were included in the study: gestational age of equal or less than 12 weeks at first visit, a BMI of between 30 and 18.5 kg/m^2^ and a blood pressure of less than 140/90mm/Hg during first visit.

The exclusion criteria are as follows: Women with a positive history of diabetes type one or two or those with a previous history of GDM, fetal macrosomia, individuals who used medications that affect carbohydrate metabolism such as glucocorticoids, patients who had diseases related to metabolism of carbohydrates such as liver and thyroid related diseases, subjects who used tobacco or alcoholic products, participants with anemia or hemoglobinopathies or hematologic diseases that affect HbA1C, those with a history of high triglyceride or cholesterol, and had multiparity pregnancies.

**Study protocol** *: *Recruitment began in April 2015 and continued up to April 2016. For the recruitment of patients, midwives were trained at all the previously mentioned medical health care centers regarding objectives of study, patient selection, follow-up of patients, and questionnaires related to the study. 

The reasons for the very careful assessment of individuals and the very specific inclusion and exclusion criteria were to ensure the inclusion of healthy patients, and to minimize any bias relating to diabetes risk factors that may have affected our primary outcome.

Primary evaluation of each patient was done by the midwifes who were stationed at each medical care center, and information regarding age of mother, gestational age, gravidity, height, weight, BMI, and blood pressure were registered. After which, each patient was referred to one of four medical laboratories affiliated to Zanjan University of Medical Sciences. Blood samples were obtained for evaluation of complete blood count (CBC), fasting blood sugar (FBS), high density lipoprotein (HDL), low density lipoprotein (LDL), triglyceride (TG), and HbA1C. In cases that any of the blood parameters were higher than the normal standard limits for the first trimester of pregnancy (9), those individuals were excluded from the study.

**Measurements: **All samples obtained were stored in oxalated tubes and kept in appropriate freezers at the medical care centers and were then transferred to Valiasr Hospital which is the central hospital in Zanjan city, Iran. For evaluating HbA1C status, the NGSP method (according to the 2016 ADA guidelines) was utilized using the Nycocard kit (Alere Inc., Austria). For the quantitative assessment of glucose levels, the Glucose GOD kit (PARS AZMUN coop., Tehran, Iran) using the photometry method, was utilized. For the evaluation of HDL, cholesterol and TG levels, the Bionik kit (Bionik, Tehran, Iran) was applied. For measurement of CBC and Hb the Sysmex XP-300™ (Japan) cell count machine was used.

**Follow-up studies: **Individuals who entered the study, were given follow-ups. At 20-24 weeks of gestational age, patients were re-examined and oral glucose tolerance test (OGTT) with 75 mg glucose was done (10). For the evaluation of glucose levels, individuals were asked to fast eight hours prior to the tests. Initially, FBS was measured, after which individuals were given 75 mg of oral glucose, and blood glucose levels were checked one hour (1 hour OGTT) and two hours (2 hour OGTT) after glucose consumption. During this stage of follow-up, HbA1C was checked for the second time, in addition systolic and diastolic blood pressures were also re-measured. All patients who were diagnosed with GDM during the 20-24 week follow-up, were referred to nutritionists and given appropriate treatments.

During a second follow-up at 24-28 weeks, all OGTT's and physical examinations were repeated, however HbA1C was not measured and in case of diagnosis of GDM based on OGTT, those individuals would then be referred to an endocrinologist for treatment. During follow-up, if a patient would have an abortion, premature labor, or intrauterine fetal death, that individual would be excluded from the study. In addition, the patients were followed-up during birth and information on pregnancy related complications such as eclampsia, bleeding, cesarean section, and premature birth was registered.

**Diagnosis:** For the diagnosis of GDM, the ADA guidelines 2016 were used. Accordingly, any patient who had FBS of more than 92mg/dl, 1 hour glucose level ≥180mg/d and 2 hour glucose level of ≥153mg/d, was considered GDM (3).

**Objectives of study:** The main goals of this registry included: 1) to evaluate the efficacy of early assessment (first trimester) of GDM according to HbA1C among women with no previously established risk factors or women with little risk factors for development of GDM, 2) to determine a cutoff point for HbA1C measured at first and second trimester of pregnancy, for the prediction of GDM.

**Data collection: **Data were collected at the mentioned health care centers and were then stored at the Metabolic Disease Research Center affiliated to Zanjan University of Medical Sciences, Zanjan, Iran. The data base was managed by a team at the research center and data entry was monitored carefully.

**Outcome measures: **Information regarding age, body mass index (BMI), systolic and diastolic blood pressure measured during three different intervals, triglyceride, cholesterol, high density lipoprotein (HDL), low density lipoprotein (LDL), hemoglobin levels (Hb), FBS, 1 hour and 2 hour OGTT tests, HbA1C, complication including number of cesarean sections, bleeding, fetal death and fetal macrosomia, were registered for each patient.

**Sample size calculation:** Sample size was calculated considering a type one error of 0.05, a p of 0.1 and an effect size of 0.03% considering HbA1C as the primary outcome. Accordingly, a sample size of 400 was needed for the study. 

**Statistical analysis:** Data were analyzed using the SPSS^®^ software for windows^®^, Version 16, (SPSS Inc., Chicago, IL, USA). 

For used the comparison of normally distributed quantitative variables between any two groups, the independent t-test was and for variables without a normal distribution, the Mann-Whitney test was employed. For comparison of qualitative variables between groups, the chi-square and Fisher's exact test were applied. 

For the comparison of data during follow-up (repeated variables) in a single group, the paired t-test and repeated measurement test were utilized. Pearson's correlation was used to evaluate the association between glucose measurement indexes (FBS, 1hour and 2 hour OGTT, and HbA1C) during all follow-ups. To predict the development of GDM during 20-24 weeks and 24-28 weeks of pregnancy, the receiver operating curve (ROC) analysis was used to estimate appropriate cutoff point based on HbA1C measured during the first trimester and 20-24 weeks of gestation, furthermore, an appropriate cutoff was also defined for FBS as well.

The logistic regression analysis was used to evaluate risk factors for the development of GDM during both follow-up periods, separately. 

According to the obtained cutoff points for HbA1C and based on previous literature, the risk factors obtained in the previous logistic regression model were transformed into categorical variables and the regression analysis was repeated to obtain relative risk (RR) for prediction of GDM. A p-value of less than 0.05 was considered statistically significant.

## Results

Initially 432 individuals were recruited for the study. During a follow-up, a total of 18 patients did not continue the follow-up and 58 cases were withdrawn from the study. In the end, 356 individuals remained in the study. As this was a cohort registry and participants were followed carefully, no missing data existed regarding any of the measured variables. Patients' baseline characteristics are shown in table 1. 

Overall, 25 individuals and 30 individuals developed GDM during 20-24 weeks and 24-28 weeks of gestational age, respectively.

**Table 1:** Comparison of baseline and clinical characteristics between those diagnosed with gestational diabetes mellitus at first trimester and 20-24 weeks of gestation and normal individuals

**Variables**	**Total ** **(n=356)**	**GDM g1** * **(n=25)**	GDM g2** **(n=30)**	**Normal ** **(n=301)**	**p-value** **(g1 and normal)**	**p-value** **(g2 and normal)**
Age	26.4 ± 4.3	27.1 ± 4.3	27.7 ± 4.2	26.3 ± 4.3	0.398	0.061
BMI	25.3 ± 3.7	27.8 ± 3.2	28.2 ± 2.8	25.1 ± 3.6	<0.001	<0.001
Systolic BP1	101.2 ±9.9	102.4 ± 9.2	105.6 ± 9	101.1 ± 10	0.389	0.011
Systolic BP2	103.6 ±1.2	113.6 ± 16.5	113.5 ± 16.5	102.8±12.2	0.002	0.001
Systolic BP3	102.1 ±1.3	114.7 ± 18.1	113.9 ± 17.3	101.1±12.7	<0.001	<0.001
Diastolic BP1	64.7 ± 8.3	66.4 ± 7.8	68.8 ± 7.5	64.5 ± 8.3	0.261	0.004
Diastolic BP2	67.8 ± 8.1	74.4 ± 10.9	74.3 ± 9	67.3 ± 7.6	<0.001	<0.001
Diastolic BP3	67.4 ± 9.1	76.2 ± 10.5	76.9 ± 10	66.7 ± 8.6	<0.001	<0.001
TG	95.1 ±33.2	101.8 ± 40	102.4 ± 32.7	94.6 ± 33.6	0.474	0.238
Cholesterol	139.8±326	150.6 ± 26.6	138.8 ± 34.2	139 ± 32.8	0.086	0.865
HDL	49.1±11.6	49.3 ± 10.7	47.2 ± 8.2	49.1 ± 11.6	0.713	0.688
LDL	76.32±2.19	80.6 ± 17.8	76.2 ± 20.2	76 ± 22.2	0.31	0.972
Hb	12.8±0.87	12.8 ± 0.6	13 ± 0.7	12.8 ± 0.8	0.713	0.167
HbA1C1	5.083±0.44	5.81 ± 0.42	5.67 ± 0.41	5.02 ± 0.38	<0.001	<0.001
HbA1C2	5.138±0.54	6.17 ± 0.65	6.17 ± 0.52	5.06 ± 0.44	<0.001	<0.001
HbA1C1≥5.3%	119 (33.4)	23 (92)	24 (80)	-	<0.001	<0.001
HbA1C1≤5.3%	237 (66.6)	2 (8)	6 (20)	-		
**Complications **						
Cesarean section	69	10 (14.5)	12 (17.4)	59 (85.5)	0.015	0.006
Bleeding	15	4 (26.7)	6 (40)	11 (73.3)	0.015	0.001
Fetal death	5	2 (40)	1 (20)	3 (60)	0.042	0.358
fetal macrosomia	28	11 (39.3)	14 (50)	17 (60.7)	<0.001	<0.001

GDM: gestational diabetes mellitus; BMI: body mass index; BP: blood pressure; TG: triglyceride; HDL: high density cholesterol; LDL: low density cholesterol; HbA1C: hemoglobin A1C

* GDM g1 are those who were diagnosed with GDM during first trimester GDM g2 are those who were diagnosed with GDM during 20-24 weeks of gestation. Furthermore, BP one, two and three show first trimester, 20-24 weeks and 24-28 weeks of gestation.

Comparison of those diagnosed with GDM during 20-24 weeks of gestational age and those diagnosed during 24-28 weeks with the normal population showed that, individuals with GDM, had significantly higher systolic and diastolic BP during both 20-24 weeks and 20-28 weeks (p<0.05). These individuals had significantly higher HbA1C at both measurements compared to that of the normal population (p<0.001). 

Those with GDM further showed a significant increase in HbA1C from the first trimester to the 20-24 weeks of follow-up (5.81%±0.42% vs. 6.17%±0.65% for the 20-24 GDM group, and 5.67%±0.41% vs. 6.17%±0.52% for the 24-28 week GDM group, p<0.001). Regarding pregnancy-related complications, those with normal pregnancies had significantly higher rates of cesarean sections (P=0.006), postpartum bleeding (P=0.001), and fetal macrosomia (p<0.001) compared to those diagnosed with GDM. In the 20-24 week diagnosed GDM group, fetal death rates were higher compared to both the normal group (p=0.042) and the 24-28 week diagnosed GDM group (20% vs. 40%) (table1).

FBS (measured during first visit), 1 hour OGTT, 2 hour OGTT, and HbA1C consecutive measurements all showed a significant increase among mothers in the study during follow-ups (table 2). Blood glucose-related measurements were correlated, results showed that HbA1C measured at first visit and at 20-24 weeks were correlated with all related glucose measurement tests (FBS, 1 hour and 2 hour OGTT during all visits, p<0.001). HbA1C at first visit was more strongly correlated with HbA1C at 20-24 weeks (r=0.639), followed by OGTT at 24-28 weeks (r=334) and OGTT at 20-24 weeks (r=0.295), respectively. HbA1C at 20-24 weeks was more correlated with HbA1C at first visit, followed by OGTT at 24-28 weeks (r=438) and 1 hour OGTT at 24-28 weeks (r=0.394), respectively (table 3).

**Table 2:** Gestational diabetes mellitus related tests during first visit and follow-up visits

**Variables**		**First trimester**	**20-24 wks**	**24-28 wks**	**p-value**
FBS		76.28 ± 6.22	79.11 ± 8.09	80.75 ± 6.8	<0.001
1hr OGTT			130 ± 28.5	132.9 ± 26.4	0.001
2hr OGTT			117.9 ± 20	119 ± 18.6	0.091
HbA1C		5.08 ± 0.44	5.13 ± 0.54		<0.001
GDM based on	FBS		14 (56)	18 (60)	
	1hr OGTT		17 (68)	17 (56.7)	
	2hr OGTT		12 (48)	13 (43.3)	
	FBS and 1hr OGTT		7 (28)	9 (30)	
	1hr OGTT and 2hr OGTT		9 (36)	8 (26.4)	
	FBS and 2hr OGTT		5 (20)	5 (16.6)	
	FBS and 1hr OGTT & 2hr OGTT		3 (12)	3 (10)	
	Overall		25 (100)	30 (100)	

FBS: fasting blood sugar; OGTT: oral glucose tolerance test; HbA1C: hemoglobin A1C; GDM: gestational diabetes mellitus

**Table 3:** Correlation between blood glucose measurement indexes during first visit and all follow-up periods

	**2hr OGTT2**	**1hr OGTT2**	**FBS3**	**HbA1C2**	**2hr OGTT1**	**1hr OGTT1**	**FBS2**	**FBS1**	**HbA1C1**
HbA1C1	0.232*	0.273*	0.334	0.639	0.280*	0.278*	0.295	0.256*	1
FBS1	0.401	0.456	0.396	0.281	0.483	0.511	0.464	1	
FBS2	0.524	0.556	0.536	0.383	0.517	0.595	1		
1hr OGTT1	0.742	0.869	0.483	0.351	0.843	1			
2hr OGTT1	0.792	0.756	0.449	0.315	1				
HbA1C2	0.327	0.394	0.438	1					
FBS3	0.273	0.582	1						
1hr OGTT2	0.824	1							
2hr OGTT2	1								

HbA1C: hemoglobin A1C; FBS: fasting blood glucose; OGTT: glucose tolerance test

* Numbers associated with values demonstrate time of measurements as followed: FBS1 measured at first trimester; FBS2 measured at 20-24 weeks of gestation, FBS3 measured at 24-28 weeks; OGTT1 measured at 20-24 weeks; OGTT2 measured at 24-28 weeks; HbA1C1 measured at first trimester; HbA1C2 measured at 20-24 weeks.

As the main outcome of the study, the ROC curve analysis showed that HbA1C during first visit (during the first trimester) at a cutoff of 5.35% can predict GDM of 24-28 weeks of pregnancy with an accuracy of 85.6%, sensitivity of 80% and specificity of 80%. For the diagnosis of GDM at 20-24 weeks of pregnancy, HbA1C at first visit at a cutoff of 5.45%, had an accuracy of 93.3%, sensitivity of 87%2, and specificity of 92%. At a cutoff of 5.75%, HbA1C measured during 20-24 weeks had an accuracy of 94.5%, sensitivity of 97%, and specificity of 96% for the diagnosis of GDM of 24-28 weeks. Furthermore, HbA1C at 20-24 weeks also showed an accuracy of 91.4%, sensitivity of 97% and specificity of 92% at a cutoff of 5.85% for diagnosis of GDM at 20-24. FBS was also used to predict GDM at 20-24 weeks and 24-28 weeks, and accordingly, a cutoff of 78.5mg/dl could estimate GDM at 24-28 weeks with an accuracy of 74.4%, sensitivity of 64% and specificity of 73%, and a cutoff of 79.5mg/dl could predict GDM at 20-24 weeks of pregnancy with an accuracy of 80.9%, sensitivity of 76% and specificity of 76%. Results also showed that OGTT diagnosed at 20-24 weeks of pregnancy had a sensitivity of 53% and specificity of 97% for the diagnosis of GDM during 24-28 weeks of follow-up.

We used a regression analysis to predict risk factors associated with GDM at both follow-ups. Results showed that two factors of BP at 20-24 weeks (beta: 3.73, P=0.001) and HbA1C at first trimester (beta: 4.167, p<0.001) were risk factors for GDM at 20-24 weeks, moreover BMI (beta: 0.184, P=0.003), BP at 20-24 weeks (beta: 1.689, P=0.019) and HbA1C at first trimester (beta: 2.25, p<0.001) were risk factors for the development of GDM at 24-28 weeks of pregnancy. When we repeated the regression analysis according to categorical variables, BMI higher and equal to 27 kg/m^2^ also presented as a risk factor (RR: 3.4, 95% CI = 1.23-9.49) for the development of GDM at 20-24 weeks of pregnancy (table 4). 

**Table 4:** Regression models for estimating risk factors of developing GDM

**Variables**	**Beta**	**RR**	**95% Confidence interval**	**p-value**
Regression model no. 1				
**20-24 wks**				
BMI	0.118	-	-	0.089
Age	0.559	-	-	0.345
BP2†	3.733	-	-	0.001
HbA1C1	4.167	-	-	<0.001
**24-28 wks**				
BMI	0.184	-	-	0.003
Age	0.60	-	-	0.242
BP2	1.689	-	-	0.019
HbA1C1	2.25	-	-	<0.001
Logistic regression no. 2				
**20-24 wks**				
BMI≥27		3.4	1.23-9.49	0.018
age≥25		1.7	0.54-5.62	0.34
BP2≥140/90		37.1	3.56-387.4	0.002
HbA1C1≥5.3%		56.6	6.95-462.3	0.001
**24-28 wks**				
BMI≥27		5.92	2.3-15.26	0.001
age≥25		1.18	0.65-5.03	0.25
BP2≥140/90		4.75	1.08-20.82	0.039
HbA1C1≥5.3%		9.1	3.38-24.64	0.001

RR: relative risk; BMI: body mass index; BP: blood pressure: HbA1C: hemoglobin A1C

* Two regression models have been introduced. The first represent independent variables as quantitative data and in the second model values have been redefined according to appropriate cut-off values obtained both in the study and from previous studies.

† Numbers indicate time of measurements as followed: BP2 as BP measured during 20-24 weeks of gestation; HbA1C1 as measured during 20-24 weeks of gestation.

## Discussion

Herein we developed a cutoff point based on HbA1C and FBS of the first trimester and 20-24 weeks of gestation among pregnant women to predict development of GDM in the settings of a cohort study. Considering the effects of high blood sugar on maternal and fetal health during pregnancy, to the best of the authors' knowledge, this is the first study that has evaluated the clinical value of early screening based on HbA1C in the setting of a cohort study among women with minimal previously known risk factor for development of GDM. We screened mothers in the first trimester of pregnancy, which was one month earlier than the guidelines reported by the ADA 2016 (11) and WHO 2013 (2), which mainly focus on pregnant woman with associated risk factors for GDM and we found that HbA1C measured at the first trimester at a cut-off of 5.35%, can predict GDM with high accuracy (>80%), moreover, HbA1C at a cut-off of 5.75%, measured during the second trimester, can predict GDM with an accuracy of more 90%. 

A total of 7% and 8.3% of individuals in our study developed GDM during 20-24 weeks and 24-28 weeks of pregnancy. In a retrospective study in 2014, Fong et al. (12) evaluated 526 woman using HbA1C of first trimester, they compared those with HbA1C of less than 5.7% and those with HbA1C of 5.7-6.4%. They found those with HbA1C of 5.7-6.4% to have a 2.4 higher chance of developing GDM. In the latter study, OGTT with 100 gr of oral glucose was used to determine GDM, more importantly, the cutoffs used for HbA1C were predetermined according to previous cutoffs in non-pregnant patients. We defined appropriate cutoff points according to our own cohort follow-up, in addition, we defined cutoff points in normal women without any to minimal previous risk factors for the development of GDM to advocate a guideline for early screening using HbA1C of first trimester among the apparently healthy pregnant women. We found that HbA1C measured at the first trimester was high among both the groups of individuals who were diagnosed with GDM during 20-24 weeks and 24-28 weeks of pregnancy. This finding is valuable as it shows that HbA1C at first trimester can be a predictor of GDM not only among high risk individuals, but among healthy mothers with minimal GDM related risk factors as well.

Our final results showed that a cut-off of 5.3% for HbA1C at first trimester was able to predict 80% of cases of GDM at 24-28 weeks of pregnancy with an accuracy of 85%. When we separately categorized individuals based on having an HbA1C of more or less than 5.3%, according to our own obtained cutoff points, both GDM groups showed a significant difference with the normal population, which supports our primary findings that perhaps 5.3% is an appropriate cutoff point to perform early screening for pregnant women. Additionally, we further evaluated the efficacy of HbA1C at 20-24 weeks of pregnancy and found that at a cut-off of 5.75%, HbA1C could predict 97% of cases of GDM of 24-28 weeks with a an accuracy of 94.5%. 

Rajput (13) evaluated the efficacy of HbA1C measured during 24-28 weeks of pregnancy for the diagnosis of GDM among 607 women. They found HbA1C at a cutoff of ≥5.95% to have an accuracy of 80.5%, sensitivity of 28.6% and specificity of 97.2% for the diagnosis of GDM. Futhermore, at a cutoff of ≥5.45%, HbA1C had a sensitivity of 85.7% and specificity of 61.1% for the diagnosis of GDM. We found a cutoff of 5.35% and 5.75% for HbA1C at first trimester and 20-24 weeks, respectively to be ideal for the diagnosis of GDM at 24-28 weeks of gestation. Comparison of the two studies, shows that perhaps HbA1C cutoff points increase with increased gestational age, and considering that insulin resistance increases throughout pregnancy (14) which consequently increases mean HbA1C, as shown in our study, this finding seems logical. Although it should be kept in mind that physiological anemia and increased blood plasma which occurs naturally during pregnancy, it can cause HbA1C to be lower at the beginning of pregnancy compared to non-pregnant women. This was also shown in the study by Hiramutsu et al. (15) in 2012, who showed that HbA1C decreases during the second trimester of pregnancy and increases during the third trimester of pregnancy. This points to the importance of defining a specific cutoff point for HbA1C during each trimester, as in our study in which we defined two different cutoff points once during the first trimester of pregnancy as an early screening tool and secondly at 20-24 weeks of gestation.

Another finding which supports the notion of an increase of insulin resistance in pregnancy was the increase in FBS which was shown to be significant during the first visit (at the first trimester) and all follow-up visits.

From another perspective, our obtained cutoff points for HbA1C for the prediction of GDM were 5.35% and 5.75%. The first cutoff point included a normal range HbA1C and the second included pre-diabetic individuals (in the definition of diabetes in normal healthy adults). This finding was similar to that of the mentioned study by Rajbut (13) who also found that at a cutoff of ≥5.45%, HbA1C had an acceptable sensitivity and specificity for diagnosis of GDM. The reason for this may be attributed to the previously mentioned physiological anemia and increased blood plasma which causes a decrease in HbA1C compared to non-pregnant women (14, 15), thus a seemingly normal HbA1C showed to be a strong predictor of GDM in our study. Another interesting finding was the cutoff point defined for FBS at first trimester, as those who had FBS of higher than 79.5mg/dl, developed GDM at 24-28 weeks of pregnancy with a sensitivity and specificity of 76% and an accuracy of 81%. This shows that mothers who have a FBS of higher than 79.5mg/dl, would benefit from an earlier screening test (for example at 20-24 weeks) for GDM. In our correlation analysis we found a positive and significant association between FBS measured at first trimester and all consecutive visits with HbA1C at first visit and at 20-24 weeks of gestation, this shows that individuals who present with high HbA1C at first visit have a concomitant high FBS.

Although we excluded individuals with most previously known risk factors for GDM such as obesity, hypertension and old age, individuals who ended up developing GDM in our study had higher BMI, and systolic and diastolic BP compared to the normal group, although all were in the normal range. On one hand, HbA1C is a means of controlling blood glucose among those with proven diabetes, on the other hand, it shows the status of glucose control during past months. Accordingly, based on our findings, development of GDM (similar to that of complication which develops due to uncontrolled BS due to GDM), may be highly affected from previous glucose control, which is then predictable with HbA1C testing. Our final regression model showed that individuals with an HbA1C of ≥5.3% at first trimester, based on our obtained cutoff point, have a 9.1 time higher risk of developing GDM during 24-28 weeks of gestation. 

This study was not without limitation. As pregnancy related complications are not very common, our sample size in the GDM groups was relatively small considering these outcomes as we calculated our sample size according to our primary outcome which was to obtain an appropriate cutoff point based on HbA1C for prediction of GDM, and comparisons of complication may not have been accurate. Like the higher recorded complications among those with normal pregnancies, which is probably attributable to the small sample sizes in the GDM groups. On the other hand, this was a cohort study and all individuals in the study were followed for the development of GDM, which renders valuable results regarding outcomes. We used the ADA guidelines for the definition of our GDM cases, however, other guideline also exists which may render different results regarding cutoff points. Our study included women who were overweight (BMI between 25 kg/m^2^), and these individuals may have had a minor risk factor for the development of GDM and would not be considered as completely without risk factor. This was also shown in our regression model which showed a BMI of more than 27 to be a significant predictor of GDM. Although this does not compromise the main objective of our study, which was to define an appropriate cutoff point according to HbA1C during the first and second trimester for predicting GDM. Considering that the Iranian guideline for obstetric screening advises all pregnant women to have FBS checked at the beginning of their pregnancy and does not separate those classified as low and high risk, in this study we showed that perhaps HbA1C measured during the first trimester may significantly aid in predicting those who may develop GDM later in their pregnancy. Moreover, we found that FBS at a cutoff of 79.5mg/dl could predict GDM at 20-24 weeks of gestation, so although screening is advised according to our country guidelines, our study adds important information on the specific cutoff point at which GDM will be predictable.

Another issue relates to the cost-effectiveness of evaluating HbA1C for all women during first trimester despite any associated risk factors. Although HbA1C during the second trimester was able to detect GDM development with more than 95% accuracy, sensitivity and specificity, we found that this method of screening during the first trimester had an accuracy of 85% and more importantly a sensitivity of specificity of 80%, meaning that 20% of individuals would not be detected on initial screening. This requires studies to be conducted focusing on the cost-effectiveness of the current result to precisely evaluate the exact implementation of these findings in clinical guidelines. 

As we did not separate those with BMI higher than 25-30 kg/m2 and those under 25Kg/m2, our cutoff points were similar for all pregnant women, even those who were overweight. As guidelines recommend OGTT during 24-28 weeks, we first performed this at 20-24 weeks. However, our diagnosis of GDM was mainly based on the OGTT test performed during 24-28 weeks which was according to the previously mentioned ADA guidelines, on the other hand we performed another OGTT test at 20-24 weeks of gestation. The main purpose of this endeavor, according to our objective of study, was to determine the value of earlier screening programs in the diagnosis of GDM both at the recommended and at an earlier time. More importantly, this does not compromise our main objective of study, which was to evaluate the efficacy of earlier screening based on HbA1C on the diagnosis of GDM. We did not consider drop-out rates, thus, the final total number of individuals who entered the study was below the calculated sample size. Fortunately, this did not affect our final outcome significance. Although recommendation to perform GDM screening in 24 weeks is mainly based on changes in blood sugar metabolism after half pregnancy, we performed our tests using HbA1C at an earlier time to show that early levels of HbA1C may be a strong predictor of GDM in the second and third months of pregnancy. In conclusion according to our findings, starting screening programs during the first trimester and at 20-24 weeks of pregnancy using HbA1C, can significantly aid in the earlier detection of GDM, even among women with minor previously known risk factors. More importantly, we found that an HbA1C of ≥5.3% in the first trimester and HbA1C of ≥5.75% at 20-24 weeks of gestation can be a good predictor of GDM with high accuracy.
